# A standardized and safe method of sterile field maintenance during intra-operative horizontal plane fluoroscopy

**DOI:** 10.1186/1754-9493-4-20

**Published:** 2010-12-13

**Authors:** Serge C Kaska

**Affiliations:** 1 488 East Valley Parkway Suite 214, Escondido, CA 92025, 760-432-6311, 760-432-0531

## Abstract

**Background:**

Intra-operative fluoroscopy for orthopaedic procedures frequently involves imaging in the horizontal plane, which requires the lower portion of the C-arm (x-ray tube) to be rotated from an unsterile zone (beneath the table) into the sterile field. To protect the integrity of the sterile field the C-arm must be draped repeatedly throughout the surgical case. The current, un-standardized, practice employs draping procedures which violate the Association of peri-Operative Registered Nurses (AORN) Standards and Recommended Practices, waste time and material, and pose an increased risk for surgical site infection.

**Presentation of the hypothesis:**

Use of a novel sterile C-arm drape (C-armor) that maintains the integrity of the sterile field, will improve operating room efficiency and reduce surgical site infection risk factors. This reduction in risk factors may potentially reduce surgical site infections in orthopaedic surgical cases requiring repeated horizontal x-ray imaging.

**Testing the Hypothesis:**

Savings in time and material and the reduction in surgical site infection risk factors afforded by using C-armor are intuitive to those skilled in the practice of orthopaedic surgery. Testing for a reduction in the number of microorganisms introduced to the surgical site by improved C-arm draping would be challenging due to the multiple confounding factors during a surgical operation. Determination of an absolute reduction in surgical site infections may be possible, but will require accounting for many confounding variables and a large study sample in order to achieve statistical significance.

**Implications of the Hypothesis:**

Improved intraoperative workflow, healthcare savings and a reduction in surgical site infection risk factors will be achieved by utilizing a standardized and safe method of sterile field maintenance during intra-operative horizontal plane fluoroscopy.

## Background

The infection rate of surgical cases is estimated at 2-3%, which likely represents an underestimation due to under-reporting [1]. Moreover, surgical site infections (SSIs) are becoming more frequent due to antibiotic resistant bacteria, and people living longer with more medical comorbidities [1]. Postoperative infection is a morbid complication, frequently leading to multiple operations, long-term use of antibiotics with their associated side effects, pain, and potential prolonged disability. Nationwide, SSIs may account for 10 billion dollars in health care expenditures annually [1]. The cost of a single surgical site infection can exceed 30,000 dollars [1,2]. Tremendous efforts to reduce hospital acquired infections are currently underway including the Surgical Care Improvement Project (SCIP) and specialty society campaigns such as the Association for Professionals in Infection Control (APIC) "Target Zero Program." Potentially preventable infections are considered avoidable by some third party payers as indicated by the policy of withholding hospital re-imbursement for some hospital acquired infections by the Centers for Medicare and Medicaid as of October 1^st ^2008, and now by Blue Cross [3,4].

Several well-known procedures that help control the risk of surgical site infection include pre-operative antibiotics, proper hair clipping methods, and proper surgical skin preparation, as well as reduction of room activity, surgical duration, and the number of sterile field breaches [4,5]. Draping of the C-arm is an inefficiency that is well known to the orthopaedic trauma and spine community and experienced by the entire operative team daily.

**Figure 1:**
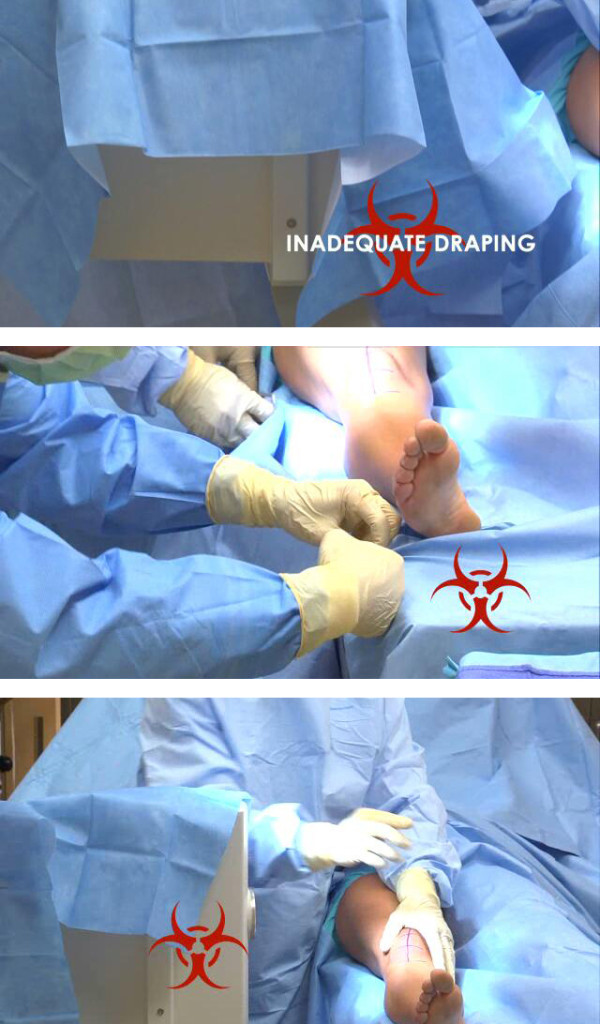
**Improvised draping techniques**. These images depict examples of common improvised draping techniques.

Since the introduction of intra-operative fluoroscopy in the 1950 s, no standard draping method or drape has been devised to protect the integrity of the sterile field while the lower portion of the C-arm (x-ray tube) repeatedly rotates from the unsterile zone into the sterile field. At present, un-standardized, inefficient, and wasteful techniques are employed for horizontal plane C-arm draping. These methods have been handed down as dogma to the last several generations of surgeons. As no other option was available, surgeons and nurses have done their best to protect patients with improvised draping techniques (Figure 1). These techniques do not adhere to AORN standards and repeatedly expose the sterile field and surgical team to contamination [6].

**Figure 2:**
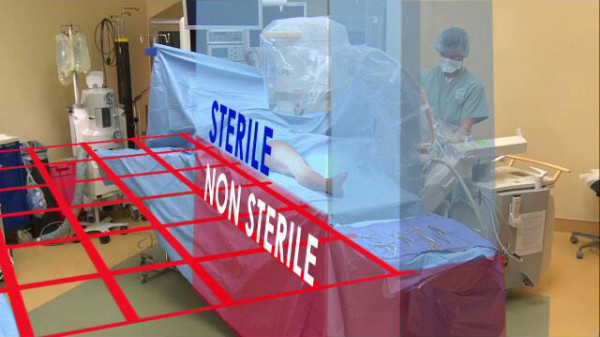
**Sterile Field**. The sterile field is defined as the horizontal plane level with the surgical tabletop as depicted by the red grid.

The sterile field is defined as the horizontal plane level with the surgical tabletop [6,7] (Figure 2). Contamination levels below the sterile field line are increased and every effort is made intra-operatively to avoid contact with this region [8]. Rotation of the C-arm into the horizontal position introduces a non-sterile object (x-ray tube) and/or contaminated drape(s) into the sterile field. This rotation is often required multiple times throughout a surgical case, which increases the potential for contamination.

The Association of peri-Operative Registered Nurses; Standards and Recommended Practices states:

*"Nonsterile equipment (eg, Mayo stands, microscopes, C-arms) should be covered with sterile barrier material(s) before being introduced to or brought over a sterile field. Only sterile items should touch sterile surfaces. The equipment should be covered with a barrier material on the top, bottom, and all sides. Sterile barrier material also should be applied to the portion of the Mayo stand or other equipment that will be positioned immediately adjacent to the sterile field." *[6]

According to the accepted definition of sterile technique, the previous standard of improvised draping breeches the sterile field and this practice should be abandoned, given the availability of cost effective new technology.

### Presentation of the hypothesis

A novel C-arm drape, which adheres to existing guidelines for sterile field maintenance will reduce risk factors linked to surgical site infection and reduce healthcare costs. The drape must provide unencumbered access to the surgical site and provide a standardized, reproducible, method of draping the x-ray tube as it enters the sterile field. The drape must also maintain its position above the sterile field line when deployed permitting maximum attention to the patient by the operative team. The drape and technique would conserve time and material by being rapidly deployable and permitting unlimited use with a single drape for each surgical case.

**Figure 3:**
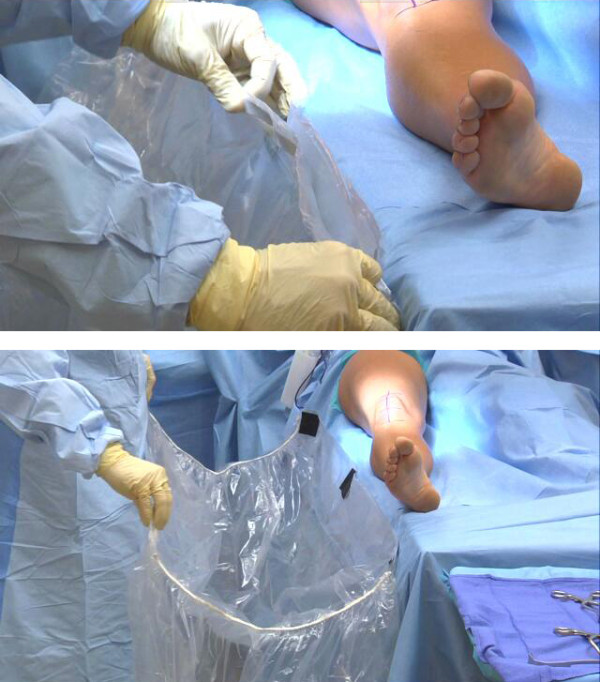
**Expandable Collapsible Drape**. The drape lies flat to the table providing unencumbered access to the surgical site, it simply manually expands with release of the fastener tabs to except the x-ray tube. The sterile pouch lies adjacent to the patient. The pouch is radiolucent allowing improved fluoroscopy targeting. The pouch also protects the x-ray tube from biohazard fluids.

**Figure 4:**
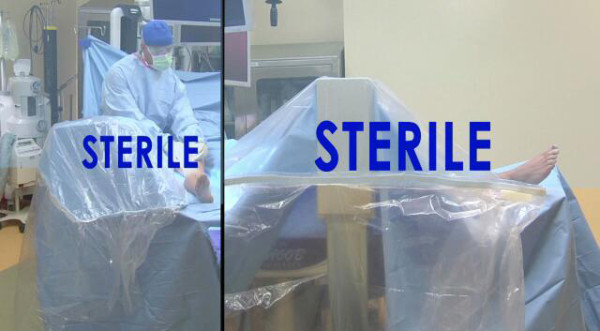
**Five sided protection**. C-armor adheres to equipment draping standards covering the x-ray tube on the top, and all four sides with a sterile barrier.

A new standardized method using a familiar technique has been developed and accomplishes the above objectives. http://www.C-armor.com The sterile pouch concept of the drape originates from the long accepted sterile pouch present in hip and arthroscopy drapes utilized for arthroplasty and arthroscopic surgery (Figure 3). Using C-armor, the non-sterile portion of the C-arm is covered on all five sides in adherence with AORN standards (Figure 4). Additional advantages include the translucent nature of the drape, which helps the surgeon and technician properly position the beam emitter for the fluoroscopy images, thus potentially reducing hazardous radiation exposure. The sterile pouch may also be used to house instruments such as the Bovie or suction tubing during the operation. The drape often further protects the sterile field by catching dropped instruments. C-armor also protects the x-ray tube from biohazard fluids. Irrigation and body fluids can not only damage the equipment but also create a means for cross-contamination from one patient to another.

### Testing the hypothesis

A direct comparison of draping techniques is impractical as no published C-arm draping techniques existed prior to the advent of C-armor. One study option is a study akin to Biswas et al., designed to swab the drape in order to measure the degree of contamination [9]. However, comparing the results to the wide array of existing techniques is not practical. Bacterial contamination of the sterile field is known to occur with time due to settling of aerosolized microorganisms [10]. It is not known, however, what degree of drape contamination is clinically significant. Furthermore a simple swab study will not take into account the potentially hazardous turbulent air flow created by improvised draping methods which moves air across the unsterile x-ray tube toward the wound or the additional risk factor reductions including: reduced operative time, and room activity afforded by C-armor.

Unpublished industry data testing the time required to drape the C-arm using the improvised methods averages 30-45 seconds (C-armor.com). The time required to deploy C-armor is 2-5 seconds (C-armor.com). Increased room activity, increased operative time, and sterile field breeches are all known SSI risk factors [1,2]. Those skilled in the practice of orthopaedic trauma and spine surgery will readily understand that C-armor will reduce operative time, room activity and the number of sterile field breeches; three known SSI risk factors. Furthermore, all previous known techniques breach the sterile field according to AORN guidelines; which has potential ethical and legal implications.

Whether the use of C-armor has a direct effect on the rate of surgical site infections is not yet known. The presumed reduction is SSIs is based on the existing literature of known risk factors and the logic that reducing these factors will lead to a reduction in infection rates. A study to definitively prove the drape reduces surgical site infections would require a very large sample size, and require accounting for many confounding variables such as medical co morbidities, degree of injury (open versus closed fractures), type of skin prep, antibiotic administration and timing, etc. Controlling for surgical technique would make it especially difficult to generate a large enough sample size. As interest and utilization grows, however, a multicenter trial could potentially gather this data. Until this data exists, utilization of equipment and techniques (C-armor) that adheres to published surgical safety principles would be prudent.

### Implications of the hypothesis

Using a standardized, efficient C-arm draping method, SSI risk factors are reduced and, hence, SSIs are likely to be reduced [1,2]. Significant reduction in red-bag waste, and operative timesavings will also occur. http://www.C-armor.com

Although the C-armor drape is a significant improvement over existing practices, several precautions must be taken to prevent fluid accumulation in the pouch and displacement of the drape below the sterile field. Blood and irrigation fluid left in the sterile pouch can be spilled onto the floor if not suctioned prior to the next horizontal plane image. When the x-ray tube is at one end of the pouch, the drape may fall below the sterile field line unless manually supported or adhered to the fastener tabs on the far end. Moreover, the drape can potentially sag below the level of the sterile field line if the existing drapes to which it is adhered are displaced in that direction intra-operatively.

C-armor is an intuitive, practical, solution to a long-standing intra-operative inefficiency and offers many intuitive benefits. As experience and technology evolve, additional improvements will be incorporated to increase patient safety and to further augment existing infection control practices at healthcare facilities.

## List of Abbreviations

AORN: Association of peri-Operative Nurses; APIC: Association for Professionals in Infection Control; SSI: Surgical Site Infection.

## Competing interests

The author currently owns intellectual property including patents and trademarks related to C-armor.
